# Multidimensional Morphology of the Ethmoid Roof and Anterior Ethmoidal Artery: A CT-Based Analysis and Proposal of the Akcan Classification

**DOI:** 10.3390/diagnostics16010081

**Published:** 2025-12-25

**Authors:** Abdullah Belada, Fatih Alper Akcan, Derya Güçlü, Ender Güçlü, İlhan Ünlü, Buğra Subaşı, Mehmet Ali Özel, Ethem İlhan, Derya Cebeci, Mehmet Ali Sungur

**Affiliations:** 1 Department of Otorhinolaryngology, Faculty of Medicine, Duzce University, Duzce 81620, Türkiyedrilhan@gmail.com (İ.Ü.); drbugrasubasi@gmail.com (B.S.); 2 Department of Otorhinolaryngology, Private Çağsu Hospital, Duzce 81600, Türkiye; f.akcan@yahoo.com; 3 Department of Radiology, Faculty of Medicine, Duzce University, Duzce 81620, Türkiye; deryasr@hotmail.com (D.G.); drmaliozel@gmail.com (M.A.Ö.); 4 Department of Otorhinolaryngology, Gaziosmanpaşa Training and Research Hospital, University of Health Sciences, Istanbul 34255, Türkiye; ethem.ilhan@sbu.edu.tr (E.İ.); drderyacebeci@gmail.com (D.C.); 5 Department of Biostatistics, Faculty of Medicine, Duzce University, Duzce 81620, Türkiye; malisungur@yahoo.com

**Keywords:** ethmoid roof, lateral lamella, anterior ethmoidal artery, paranasal sinuses, skull base, computed tomography, anatomical variation

## Abstract

**Background/Objectives**: Anatomical variation in the ethmoid roof and lateral lamella play an important role in anatomical vulnerability during endoscopic sinus and skull base surgery. However, widely used classifications, including the Keros system, primarily focus on vertical depth and may not fully reflect the complex geometric relationship between the ethmoid roof, lateral lamella, and the anterior ethmoidal artery (AEA). This study aimed to characterize ethmoid roof and lateral lamella anatomy using high-resolution CT and to propose a descriptive radiological framework—the Akcan Classification—that integrates AEA exit patterns with multiple morphometric parameters. Given the complexity of thin skull base structures, interobserver reproducibility of all morphometric parameters was additionally assessed to ensure measurement robustness. **Methods**: High-resolution paranasal sinus CT scans from 175 adults (350 sides) were retrospectively evaluated. Measurements included ethmoid roof width, lateral lamella depth, anterior–posterior length, lamellar angle, AEA–lamella distance, and sinonasal anatomical variations. Interobserver reliability was quantified using ICCs. AEA morphology was categorized as in-canal (Type 1), partially suspended (Type 2), or fully suspended (Type 3) based on radiological appearance of bony canalization. Appropriate statistical tests were used to compare morphometric features across groups. **Results**: Suspended AEA configurations demonstrated progressively wider ethmoid roofs, deeper lateral lamellae, steeper lamellar inclination, and shorter AEA–lamella distances (all *p* < 0.001). Supraorbital ethmoid cells were more frequently observed in Type 3 cases (*p* < 0.001). Other anatomical variations showed no significant association with ethmoid roof morphology. Interobserver reliability was excellent for all measurements (ICC range 0.87–0.94). **Conclusions**: The findings suggest that AEA configuration is associated with broader patterns of ethmoid roof and lateral lamella morphology. Rather than serving as a validated predictor of surgical outcomes, the Akcan Classification provides a structured anatomical and radiological descriptor that complements depth-based systems such as the Keros classification. The high reproducibility of measurements supports its potential utility for standardized anatomical assessment and preoperative radiological interpretation, while further studies incorporating surgical correlation are required.

## 1. Introduction

Anatomical variability within the paranasal sinuses represents one of the most critical factors influencing the risk of complications during endoscopic sinus and skull base surgery. Among these structures, the ethmoid roof and the lateral lamella constitute the most vulnerable regions due to their extremely thin bony architecture and the passage of the anterior ethmoidal artery (AEA) through this area [1,2]. Even minimal variations in the morphology of this region may predispose patients to severe complications, including orbital hematoma, cerebrospinal fluid leakage, and iatrogenic skull base injury [3,4]. For this reason, detailed morphometric assessment of the ethmoid roof is essential for improving anatomical understanding and for increasing awareness of anatomical vulnerability during surgical planning, rather than relying on a single parameter alone. Moreover, because skull base structures are extremely thin, small discrepancies in radiological interpretation may result in significant differences in perceived surgical risk, highlighting the need for reproducible and standardized morphometric measurements.

The first systematic classification of ethmoid roof height and lateral lamella depth was introduced by Keros in 1962, and this framework has long served as the primary reference for preoperative risk evaluation [5,6]. However, with advances in high-resolution imaging, it has become evident that vertical depth alone does not fully capture the complexity of ethmoid roof anatomy. Parameters such as ethmoid roof width, slope, anterior–posterior extension, and the course of the AEA have been shown to influence the anatomical configuration of the anterior skull base and may contribute to variations in perceived surgical difficulty [5,7]. Contemporary studies highlight that the AEA may be located within a complete bony canal, partially dehiscent, or entirely suspended beneath the skull base, and these patterns significantly modify the likelihood of intraoperative arterial injury [8,9,10].

Several classification systems have been proposed to describe AEA morphology or ethmoid roof anatomy, including the Yenigun, Floreani, and Moon classifications; however, these systems typically focus on either vertical depth or arterial canalization in isolation and do not integrate AEA configuration with a comprehensive set of ethmoid roof morphometric parameters [11,12,13,14,15]. Despite this, no existing classification correlates AEA morphology with comprehensive ethmoid roof measurements in a unified anatomical model. Furthermore, increasing evidence suggests that the spatial distance between the AEA and the lateral lamella, as well as the inclination angle of the lamella, may serve as additional anatomical descriptors of skull base configuration—parameters that are not incorporated into traditional systems [15,16,17].

Accordingly, there is a growing need for multidimensional approaches that evaluate the ethmoid roof not solely through a single morphometric parameter but rather through an integrated set of measurements, including width, depth, slope, anterior–posterior length, and the positional relationship between the AEA and the lateral lamella. Although previous research has analyzed lateral lamella height or AEA configuration separately, the literature lacks a framework that examines these two critical structures together [18,19,20]. In addition, anatomical variations such as supraorbital ethmoid cells, agger nasi cells, concha bullosa, and frontal recess cells may influence ethmoid aeration patterns and modify skull base geometry, yet their contribution to AEA exposure remains insufficiently defined. In particular, supraorbital ethmoid cells have been reported to alter the course and canalization of the AEA, potentially increasing arterial exposure beneath the skull base [21,22,23]. Clarifying these relationships may improve the consistency of radiological interpretation rather than serving as a direct predictor of surgical outcomes [1,24].

In the present study, high-resolution computed tomography was used to evaluate ethmoid roof width, lateral lamella depth, roof slope, anterior–posterior extension, and the spatial relationship between the AEA and the lateral lamella. Interobserver reproducibility of these measurements was assessed to ensure methodological robustness and to support the validity of the proposed radiological and anatomical framework. Based on these measurements, a new classification system—the Akcan Classification—was proposed, integrating AEA exit patterns with comprehensive ethmoid roof morphology. Rather than aiming to replace existing systems, this classification is intended to complement depth-based frameworks such as the Keros classification by providing a multidimensional anatomical description [25]. By combining multiple morphometric dimensions with AEA configuration and adjacent anatomical variations, the present study seeks to address a critical gap in current skull base anatomical assessment models.

## 2. Materials and Methods

### 2.1. Study Design and Ethical Considerations

This study was planned as a retrospective observational analysis performed at a tertiary otorhinolaryngology and skull base surgery center. The investigation relied exclusively on previously acquired paranasal sinus computed tomography (CT) scans without any change in imaging protocols or clinical management. The study protocol received approval from the institutional clinical research ethics committee, and all procedures were conducted according to the principles of the Declaration of Helsinki. Because the research involved only image review and no direct contact with patients, the requirement for informed consent was waived by the ethics board. Ethical approval was obtained from the Düzce University Non-Interventional Clinical Research Ethics Committee (Decision Date: 20 July 2020, Decision No: 2020/165).

### 2.2. Study Population

The study population consisted of adult patients who underwent paranasal sinus CT for diagnostic evaluation of sinonasal complaints, including facial pressure, nasal obstruction, or suspicion of anatomical variation. The inclusion of symptomatic patients reflects routine clinical imaging practice and was chosen to ensure adequate visualization of sinonasal anatomy under real-world diagnostic conditions; no assumptions were made regarding direct extrapolation to the general asymptomatic population. All examinations performed between January 2019 and June 2020 were retrospectively screened through the institutional Picture Archiving and Communication System (PACS). From this archive, 175 patients met the eligibility criteria and were included. Both right and left sides were evaluated independently, resulting in a total of 350 anatomical units.

Eligible patients were required to be at least 18 years of age and have high-resolution CT images with complete visualization of the ethmoid roof, fovea ethmoidalis, cribriform plate, lateral lamella, and the expected course of the anterior ethmoidal artery (AEA). Patients with a history of sinonasal or skull base surgery were excluded to eliminate the risk of altered anatomy, as were those with sinonasal tumors, fractures involving the nasal or ethmoid bone, invasive fungal disease, or congenital craniofacial anomalies. CT scans in which extensive sinonasal polyposis obscured critical landmarks or in which image quality was inadequate for precise measurements were also excluded.

Inadequate image quality was defined a priori as the presence of motion artifacts, incomplete visualization of the anterior skull base, or slice thickness exceeding 1.0 mm, which could compromise accurate morphometric assessment.

### 2.3. CT Acquisition Protocol

All CT examinations were performed using a 64-slice multidetector CT unit with thin-section acquisition optimized for paranasal sinus evaluation. Patients were positioned supine with the head in a neutral orientation, and images were obtained from the hard palate to the roof of the frontal sinus. The protocol used a slice thickness between 0.5 and 1.0 mm to ensure optimal spatial resolution of the skull base. A tube voltage of 120 kVp and tube current between 100 and 200 mA were applied, adjusted automatically based on patient size. A 512 × 512 matrix and a bone algorithm were used for reconstruction to enhance the delineation of thin bony structures such as the lateral lamella and ethmoid roof.

Multiplanar reconstructions (MPRs) were routinely generated in axial, coronal, and sagittal planes. Coronal images perpendicular to the hard palate were particularly critical for evaluating the fovea ethmoidalis and the vertical height of the lateral lamella, while sagittal reconstructions provided superior visualization of the anterior–posterior extent of the ethmoid roof and the relationship of the AEA to the skull base. Axial reconstructions were used to follow the orbital ethmoidal groove and identify the point at which the AEA traversed from the orbit into the ethmoid roof.

Although CT is considered the standard modality for evaluating ethmoid roof anatomy, subtle bony dehiscence—especially of the AEA—may occasionally be underestimated depending on slice thickness and reconstruction parameters. This limitation was taken into account during image interpretation and is addressed in the Discussion.

**Figure 1:**
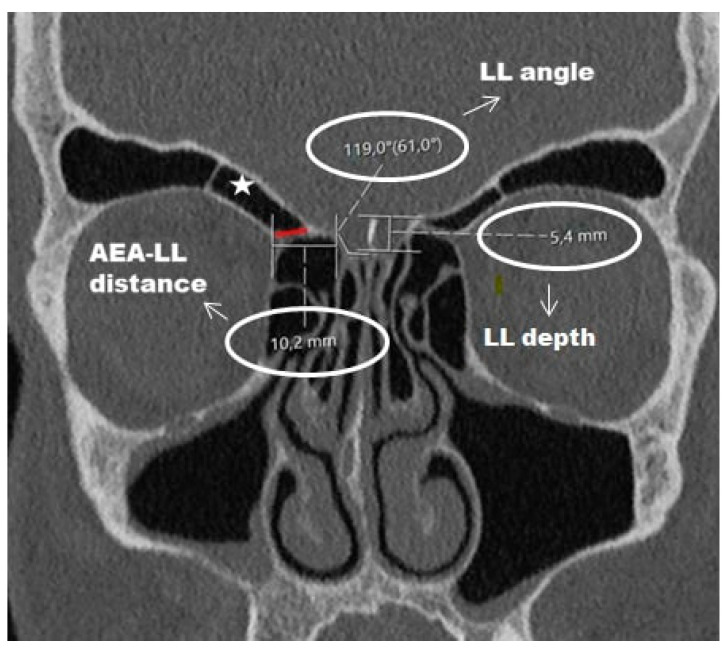
Representative coronal CT image demonstrating key morphometric measurements of the ethmoid roof and lateral lamella.

The morphometric measurements of the ethmoid roof and lateral lamella are described in Figure 1.

The figure illustrates the measurement of the lateral lamella depth, lateral lamella angle, and the anterior ethmoidal artery–lateral lamella distance (AEA–LL distance). The asterisk indicates a supraorbital ethmoid cell, while the red line marks the anterior ethmoidal artery.

### 2.4. Radiological Evaluation and Measurement Procedure

All CT studies were reviewed on a dedicated PACS workstation equipped with three-plane synchronized viewing and calibrated digital calipers. All measurements were independently performed by two otolaryngologists (A.B. and F.A.A.), both of whom have specific experience in sinonasal radiology and skull base imaging. Both observers were blinded to patient demographics, clinical indications, and each other’s measurements during the evaluation process. Each reviewer evaluated all reconstructed planes without any knowledge of the other’s results. After completion of independent review, all measurements showing discrepancy were re-evaluated together, and a consensus value was recorded. This approach ensured internal consistency and minimized observer-dependent variation.

Measurements were always taken at the level where the anatomical structure appeared best defined. Window width and level were adjusted as needed for optimal visualization of both bony and soft-tissue interfaces. Care was taken to ensure that all linear measurements were obtained perpendicular to the anatomical axis of interest to avoid angular distortion.

To assess interobserver reliability, the Intraclass Correlation Coefficient (ICC) was calculated for all primary morphometric measurements, including ethmoid roof width, lateral lamella depth, lateral lamella angle, anterior–posterior roof length, and AEA–lamella distance. ICC values were computed using a two-way mixed-effects model with absolute agreement to ensure methodological robustness. ICC values demonstrated excellent agreement between the two reviewers (ICC range: 0.87–0.94), confirming the high reproducibility and reliability of the measurement protocol.

### 2.5. Ethmoid Roof and Lateral Lamella Measurements

The ethmoid roof and lateral lamella were measured systematically on coronal and sagittal planes. Ethmoid roof width was defined as the mediolateral distance between the right and left fovea ethmoidalis. Lateral lamella depth was measured as the vertical distance between the most superior point of the cribriform plate and the inferior surface of the fovea ethmoidalis. Because subtle variations in head posture can influence the perceived height, all measurements were taken with reference to the hard palate and skull base orientation.

The anterior–posterior length of the ethmoid roof was assessed on sagittal reconstructions by identifying the anterior and posterior insertion points of the lateral lamella along the ethmoid roof. The inclination of the lateral lamella was determined by calculating the angle formed between the lamella and the horizontal plane of the anterior skull base. These parameters were selected to provide a multidimensional radiological description of skull base geometry rather than to serve as direct predictors of surgical outcomes.

### 2.6. Assessment of the Anterior Ethmoidal Artery

The AEA was examined by following its course from the frontoethmoidal suture through the orbit to the ethmoid roof using axial sections and confirming its vertical position using coronal reconstructions. The artery was identified as a thin bony canal, a partially dehiscent canal, or a completely suspended configuration adjacent to the ethmoid roof.

Each AEA was classified according to the newly proposed Akcan Classification. In this system, Type 1 represented a fully intrabony, in-canal artery embedded within the frontoethmoidal groove. Type 2 (partially suspended) was defined as partial loss of bony canalization with persistent contact between the artery and the skull base, whereas Type 3 (fully suspended) was defined as complete circumferential absence of bony canalization with the artery freely coursing beneath the ethmoid roof without bony attachment. For each artery, the shortest linear distance to the lateral lamella insertion point was measured, providing a quantitative assessment of arterial proximity to the skull base.

### 2.7. Assessment of Additional Sinonasal Anatomical Variations

Several commonly encountered sinonasal anatomical variations were also documented because of their known influence on ethmoid aeration patterns and potential relationship with morphometric differences. The presence of agger nasi cells, supraorbital ethmoid cells, concha bullosa, frontal recess cells, and septal deviation was assessed for each patient using coronal and sagittal reconstructions. Variations were assessed bilaterally but recorded per patient as present or absent to ensure analytical consistency. Variations were recorded dichotomously as present or absent, without further subclassification, to maintain consistency and avoid unnecessary complexity in categorization.

### 2.8. Statistical Analysis

All statistical analyses were performed using IBM SPSS Statistics (Version 26.0, IBM Corp., Armonk, NY, USA). Continuous variables were assessed for normality using the Kolmogorov–Smirnov and Shapiro–Wilk tests. Normally distributed variables were expressed as mean ± standard deviation (SD). Categorical variables were presented as frequencies and percentages.

Comparisons between right and left sides for continuous morphometric measurements were performed using paired-samples *t*-tests. Although measurements were obtained bilaterally, side-based analysis was chosen because ethmoid roof and AEA anatomy may exhibit clinically relevant asymmetry; this approach has been adopted in prior radiological morphometric studies. Differences among the three Akcan Classification groups were evaluated using one-way analysis of variance (ANOVA), followed by Bonferroni post hoc testing. Correlations between continuous variables were examined using Pearson correlation analysis. A two-tailed *p*-value < 0.05 was considered statistically significant.

A priori power analysis was performed using G*Power 3.1.9.7 (Heinrich-Heine-Universität, Düsseldorf, Germany). Based on one-way ANOVA with three groups, an assumed medium effect size (f = 0.25), alpha error probability of 0.05, and 80% statistical power, the minimum required sample size was calculated as 132 patients. With a final sample of 175 patients (350 sides), the study achieved sufficient statistical power (>0.80) to detect clinically meaningful differences across Akcan Classification groups [11].

## 3. Results

A total of 175 patients (350 sides) were included in the analysis. The mean age was 38.6 ± 13.9 years (range: 18–79), and 58.3% of the cohort was male. Septal deviation was observed in 64.0% of patients, followed by agger nasi cells in 50.8%, supraorbital ethmoid cells in 34.8%, concha bullosa in 33.1%, and frontal recess cells in 21.1%. Detailed baseline characteristics are presented in Table 1.

Interobserver reliability demonstrated excellent agreement across all primary morphometric parameters, including ethmoid roof width, lateral lamella depth, anterior–posterior roof length, lateral lamella angle, and AEA–LL distance. Intraclass correlation coefficients (ICC) ranged from 0.87 to 0.94, with all *p*-values < 0.001. Results are shown in Table 2.

Bilateral morphometric measurements of the ethmoid roof and lateral lamella revealed no significant differences between right and left sides. Ethmoid roof width (*p* = 0.214), LL depth (*p* = 0.482), AP roof length (*p* = 0.331), LL angle (*p* = 0.289), and AEA–LL distance (*p* = 0.567) were statistically comparable. Detailed values are provided in Table 3.

Evaluation of the Keros classification demonstrated that 24.6% of sides were Type 1, 58.0% were Type 2, and 17.4% were Type 3. No significant side predominance was observed (*p* = 0.621). The distribution is shown in Table 4.

According to the Akcan Classification, 40.0% of sides were categorized as Type 1 (in-canal), 41.7% as Type 2 (partially suspended), and 18.3% as Type 3 (fully suspended). No significant right–left difference was detected (*p* = 0.774). Results are summarized in Table 5.

Morphometric comparison across Akcan types demonstrated statistically significant differences for all primary measurements. Ethmoid roof width (*p* < 0.001), LL depth (*p* < 0.001), LL angle (*p* = 0.001), AP roof length (*p* = 0.004), and AEA–LL distance (*p* < 0.001) differed significantly among the three groups. Bonferroni post hoc analysis indicated that each Akcan type differed significantly from the others for all continuous variables (all *p* < 0.05). The data are provided in Table 6.

Analysis of the relationship between anatomical variations and Akcan types revealed that only supraorbital ethmoid cells showed significant differences across Akcan groups (*p* < 0.001). No significant associations were observed for agger nasi cells (*p* = 0.214), concha bullosa (*p* = 0.388), frontal recess cells (*p* = 0.472), or septal deviation (*p* = 0.531). The distribution is summarized in Table 7.

A statistically significant association was observed between Akcan and Keros classifications (χ^2^, *p* < 0.001). Akcan Type 3 showed the highest proportion of Keros Type 3. The cross-distribution is presented in Table 8. A statistically significant association was found between Akcan Type 3 and Keros Type 3, suggesting that increased vertical depth may accompany higher degrees of arterial suspension.

**Figure 2:**
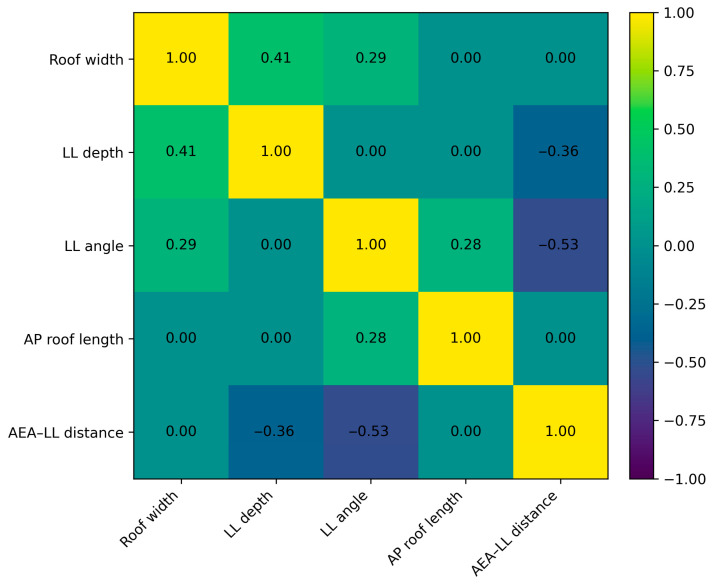
Correlation matrix heatmap of ethmoid roof and lateral lamella morphometric parameters.

Pearson correlation analysis demonstrated significant associations among morphometric variables. Ethmoid roof width correlated positively with LL depth (r = 0.41, *p* < 0.001) and LL angle (r = 0.29, *p* = 0.002). LL depth correlated negatively with AEA–LL distance (r = −0.36, *p* < 0.001). LL angle correlated negatively with AEA–LL distance (r = −0.53, *p* < 0.001). AP roof length correlated positively with LL angle (r = 0.28, *p* = 0.004). Full correlations are listed in Table 9. The overall correlation structure and the relative strength and direction of these associations are visually summarized in Figure 2 as a correlation matrix heatmap.

The heatmap illustrates Pearson correlation coefficients (r) among continuous morphometric variables, including ethmoid roof width, lateral lamella (LL) depth, LL angle, anterior–posterior (AP) roof length, and AEA–LL distance. Color intensity reflects the strength and direction of correlations. Abbreviations: AP, anterior–posterior; AEA, anterior ethmoidal artery; LL, lateral lamella.

According to Table 10, gender-based comparison of ethmoid roof and lateral lamella morphometric parameters did not reveal any statistically significant differences between male and female patients. Ethmoid roof width, lateral lamella depth, anterior–posterior roof length, lateral lamella angle, and AEA–lateral lamella distance were comparable between sexes (all *p* > 0.05). These findings suggest that, within the studied adult population, sexual dimorphism did not have a measurable impact on the morphometric characteristics of the ethmoid roof or the spatial relationship of the anterior ethmoidal artery.

Values are presented as mean ± standard deviation (SD). Comparisons between male and female patients were performed using the independent-samples *t*-test. No statistically significant gender-based differences were observed for any morphometric parameter (all *p* > 0.05). AP = Anterior–posterior; AEA = Anterior ethmoidal artery; LL = Lateral lamella.

According to Table 11, multivariate logistic regression analysis was performed to evaluate whether the Akcan Classification remained associated with ethmoid roof morphology after adjustment for Keros classification. After controlling for vertical skull base depth, Akcan Type 3 (fully suspended AEA) remained independently associated with increased ethmoid roof width, greater lateral lamella depth, steeper lateral lamella angle, and shorter AEA–lateral lamella distance (all *p* < 0.01). These results indicate that the Akcan Classification provides complementary anatomical information beyond vertical depth alone and captures additional aspects of anterior skull base geometry not fully explained by the Keros system.

Multivariate logistic regression analysis was performed to evaluate whether the Akcan Classification (Type 3 vs. Type 1–2) remained independently associated with morphometric parameters after adjustment for Keros classification. Odds ratios (ORs) are presented with 95% confidence intervals (CI). The analysis demonstrated that Akcan Type 3 remained significantly associated with multiple ethmoid roof parameters independent of Keros depth, indicating that the Akcan Classification provides complementary anatomical information beyond vertical skull base depth alone. AEA = Anterior ethmoidal artery; LL = Lateral lamella.

## 4. Discussion

This study provides a comprehensive assessment of the ethmoid roof and lateral lamella by integrating multiple morphometric parameters—roof width, lateral lamella depth, anterior–posterior extension, lamellar inclination, and the spatial relationship of the anterior ethmoidal artery —together with common sinonasal variations. Rather than proposing a direct predictor of surgical complications, the results highlight that the anatomical vulnerability of the anterior skull base cannot be fully characterized by Keros depth alone and that a broader, multidimensional anatomical perspective may improve preoperative anatomical awareness. The observed associations between AEA morphology, ethmoid roof configuration, and aeration patterns of adjacent structures offer new insights into the structural heterogeneity of the anterior skull base. In addition, the excellent interobserver reliability achieved for all morphometric parameters suggests that these measurements can be reproduced consistently, supporting their potential applicability in descriptive radiological evaluation.

Ethmoid roof width showed substantial interindividual variation and demonstrated a progressive increase from Akcan Type 1 to Type 3 configurations. A wider roof may reflect expanded ethmoidal aeration, potentially accompanied by attenuation of the bony support surrounding the AEA [26,27]. From an anatomical perspective, increased width may alter the position of key landmarks and modify the geometry of the frontoethmoidal transition zone, which is relevant during dissections involving the frontal recess, superior turbinate, or medial orbital wall. Importantly, this association should be interpreted as a morphologic pattern rather than a direct indicator of operative risk, as the relationship between roof width and arterial canal integrity has not yet been validated intraoperatively. The positive correlation between roof width and lateral lamella depth further suggests that cranial base expansion may occur as part of a broader morphologic configuration rather than as an isolated anatomical variant [28,29].

Lateral lamella depth, traditionally described through the Keros classification, remains one of the most clinically relevant skull base measurements. Consistent with previous reports, deeper lamellae were more frequently associated with suspended AEA patterns [5,30]. However, the present findings indicate that depth alone may not sufficiently capture the complexity of anterior skull base anatomy. The combined evaluation of lamellar depth, inclination, and anterior–posterior extension suggests that structural vulnerability is more accurately conceptualized as a geometric interaction of multiple parameters rather than a single vertical measurement. The observed negative correlation between lateral lamella depth and AEA–lamella distance supports this interpretation and underscores the importance of evaluating spatial relationships alongside depth measurements.

The anterior–posterior length of the ethmoid roof, a parameter rarely discussed in the literature, showed meaningful variation and appeared to correlate with AEA exposure. A longer ethmoid roof may reflect a more posteriorly positioned lateral lamella insertion, which in turn elongates the transition zone between the frontal and ethmoid sinuses [26,31]. While this finding does not independently define high-risk anatomy, its correlation with lamellar angle suggests that anterior–posterior extension contributes to the overall three-dimensional geometry of the skull base when considered alongside other parameters.

Similarly, the inclination of the lateral lamella increased stepwise across the Akcan Classification. A steeper lamellar angle narrows the spatial corridor between the middle turbinate attachment and the skull base and may complicate anatomical orientation during endoscopic navigation. Rather than implying increased complication risk, these findings suggest that lamellar angle represents an additional geometric feature that may influence intraoperative spatial perception, particularly in anatomically complex ethmoid configurations. The strong inverse relationship between lamellar angle and AEA–lamella distance further supports the concept that arterial exposure occurs within a broader architectural context.

The distance between the AEA and the lateral lamella emerged as one of the most sensitive indicators of arterial exposure. Shorter AEA–LL distances were consistently associated with suspended arterial configurations. Although reduced distances have been linked to arterial vulnerability in prior anatomical and clinical reports, the present study emphasizes this measurement as a descriptive spatial parameter rather than a surrogate for operative risk [32,33]. Variations in CT reconstruction and individual skull base curvature should be considered when interpreting absolute distance values.

Supraorbital ethmoid cells demonstrated a notable association with AEA suspension. The expansion of supraorbital cells into the anterior cranial fossa may alter regional bone thickness and redirect ethmoidal aeration patterns in a manner that disrupts the bony canal of the AEA [23,34]. This finding supports the concept that specific aeration patterns—particularly those extending superiorly toward the cranial base—may act as modifiers of arterial configuration, whereas more common variations such as agger nasi cells, concha bullosa, or frontal recess cells appear to primarily influence sinonasal ventilation rather than skull base architecture [1,35].

The Akcan Classification proposed in this study offers a structured system that integrates AEA morphology with multiple quantitative ethmoid roof parameters. By linking arterial configuration not only to roof width and lateral lamella depth but also to lamellar inclination, anterior–posterior ethmoid roof length, and the spatial distance between the AEA and the lateral lamella, this classification extends beyond traditional single-parameter approaches. In this context, the Akcan Classification is designed to complement existing frameworks such as the Keros system, which primarily focuses on vertical skull base depth. The inclusion of additional geometric parameters allows a more comprehensive description of anterior skull base architecture and provides contextual anatomical information that is not fully captured by depth-based classifications alone. Although the observed associations suggest potential relevance for preoperative imaging assessment, the present findings should be regarded as preliminary, as the classification has not yet been correlated with intraoperative findings or surgical outcomes.

An additional noteworthy finding of the present study was the significant association between the Akcan Classification and the traditional Keros system, indicating that AEA exposure does not occur independently of vertical skull base depth. While the Keros classification has long served as the principal reference for estimating skull base vulnerability based on lateral lamella height, multivariate analysis demonstrated that Akcan Type 3 remained significantly associated with several morphometric parameters after adjustment for Keros depth, suggesting that the two systems capture overlapping but not identical aspects of anterior skull base anatomy. In particular, the co-occurrence of increased lamellar depth, steeper lamellar angle, shortened AEA–lamella distance, and greater anterior–posterior roof extension within Akcan Type 3 configurations supports the concept that skull base vulnerability reflects a combined geometric pattern rather than vertical depth alone. Previous studies have emphasized the increased susceptibility associated with Keros Type 3 due to the pronounced vertical drop of the cribriform plate; the present findings add to this perspective by demonstrating that arterial exposure is further influenced by lamellar orientation and spatial proximity, reinforcing the value of a multidimensional anatomical framework.

Additionally, based on imaging observations, narrower cranial configurations appeared to coincide with a narrower lateral lamella corridor and a lower prevalence of supraorbital ethmoid cells. Although this relationship was not directly quantified in the present analysis, it may help explain why suspended AEA configurations were more frequently observed in anatomically compact anterior skull base morphologies. This observation raises the possibility that global cranial geometry and local ethmoidal pneumatization patterns interact to shape AEA configuration, a hypothesis that warrants further investigation using cranial width measurements and volumetric approaches.

Several classification systems have previously been proposed to describe anterior ethmoidal artery anatomy and skull base vulnerability, most notably the Keros classification and AEA-focused systems such as those described by Floreani and Moon [6,13,14]. The Keros system primarily characterizes vertical skull base depth based on lateral lamella height and remains widely used for estimating the risk of skull base injury. In contrast, AEA-oriented classifications mainly focus on the presence or absence of a bony canal and the degree of arterial dehiscence, without integrating the surrounding ethmoid roof geometry. While these approaches provide valuable information, they evaluate isolated anatomical features rather than the broader structural context in which the AEA is situated.

The Akcan Classification differs conceptually by integrating AEA configuration with multiple quantitative parameters of ethmoid roof morphology, including roof width, anterior–posterior extension, lamellar inclination, and AEA–lamella distance. Rather than proposing an alternative to existing systems, this framework aims to contextualize arterial exposure within the overall geometry of the anterior skull base. In this regard, the Akcan Classification may be considered complementary to both depth-based and canal-based systems, as it captures geometric relationships that are not addressed by vertical height or canal integrity alone. This integrated perspective may help explain why similar Keros types or canal configurations can be associated with differing degrees of arterial exposure and perceived surgical vulnerability.

This study has several limitations. Its retrospective design limited control over imaging protocols, and variations in image quality may have influenced the visualization of thin bony structures, particularly in regions with partial volume effects. Despite excellent interobserver reliability, certain assessments—especially the evaluation of AEA canal integrity and partial dehiscence—remain subject to interpretative variability inherent to CT-based analysis. In addition, all measurements were derived from two-dimensional CT reconstructions; while the term “multidimensional” reflects the integration of multiple morphometric parameters, true three-dimensional segmentation, volumetric analysis, or spatial modeling was not performed, and such approaches may provide a more refined representation of skull base geometry in future studies.

Although analyses were conducted on a side-based level, no statistically significant right–left differences were observed across any morphometric parameter, supporting the treatment of each side as an independent anatomical unit, consistent with prior CT-based morphometric studies. Nevertheless, the absence of mixed-effects or generalized estimating equation modeling should be acknowledged as a methodological limitation.

The study population consisted exclusively of adult patients undergoing CT for sinonasal symptoms, which may introduce selection bias and limit extrapolation to asymptomatic populations or pediatric cohorts, whose ethmoid development and skull base anatomy differ substantially. Furthermore, the single-center design restricts assessment of potential ethnic or regional anatomical variability.

The Akcan Classification was developed and evaluated within a single dataset, and no internal split-sample or external validation cohort was available, raising the possibility of limited generalizability. In addition, although qualitative criteria were applied to distinguish partially suspended and fully suspended AEA configurations, quantitative threshold values separating these categories were not formally defined, which may affect reproducibility across institutions and imaging platforms. Finally, the absence of intraoperative correlation precludes direct validation of the proposed classification against surgical findings or complication rates. Accordingly, the Akcan Classification should currently be regarded as an anatomical and radiological descriptive framework rather than a validated predictor of surgical risk.

Overall, the findings of this study underscore the complex and interconnected nature of anterior skull base anatomy. By integrating ethmoid roof geometry, lateral lamella characteristics, anterior–posterior extension, lamellar inclination, AEA spatial proximity, and associated aeration patterns, the Akcan Classification provides a comprehensive and structured anatomical context that may enhance the interpretation of preoperative imaging beyond depth-based systems alone. Rather than replacing existing classifications, this approach offers complementary anatomical information that may support surgical orientation and anatomical awareness. With further validation through prospective, multicenter investigations incorporating three-dimensional modeling and intraoperative correlation, this multidimensional framework may contribute to more refined anatomical assessment and informed surgical planning in endoscopic sinus and skull base procedures.

## 5. Conclusions

In conclusion, this study demonstrates that the morphology of the ethmoid roof and lateral lamella is shaped by a complex interaction of structural parameters, including roof width, lamellar depth, anterior–posterior extension, lamellar inclination, and the spatial proximity of the anterior ethmoidal artery. Suspended AEA configurations were consistently associated with wider ethmoid roofs, deeper and more steeply angled lateral lamellae, and a marked reduction in AEA–lamella distance, reinforcing the concept that arterial exposure reflects broader skull base architecture rather than an isolated anatomical variation. Supraorbital ethmoid cells emerged as a key modifier of AEA configuration, whereas other sinonasal variations and septal deviation did not demonstrate a meaningful influence on skull base morphology. The significant interrelationships observed among morphometric parameters further emphasize the interconnected and multidimensional nature of anterior skull base anatomy.

Although the Akcan Classification offers a structured and integrative framework for describing AEA-related skull base morphology, its role at present should be considered primarily anatomical and radiological. The classification has not yet been validated against intraoperative findings, complication rates, or navigation-assisted surgical outcomes, and therefore should not be interpreted as a definitive predictor of procedural risk. Rather, it may serve as a complementary descriptive system that enhances anatomical awareness during preoperative imaging assessment alongside established classifications such as the Keros system.

Future studies incorporating multicenter cohorts, quantitative threshold definitions, three-dimensional reconstructions, and direct surgical correlation will be essential to determine whether this multidimensional approach can meaningfully enhance anatomical risk assessment and intraoperative decision-making. With appropriate validation, the Akcan Classification may contribute to more refined preoperative interpretation and support safer endoscopic management of the anterior skull base.

## Figures and Tables

**Table 1:** Baseline Characteristics of the Study Population (n = 175).

Variable	Value
Age, years (mean ± SD)	38.6 ± 13.9
Sex, n (%)	Male 102 (58.3%), Female 73 (41.7%)
Septal deviation, n (%)	112 (64.0%)
Agger nasi cell, n (%)	89 (50.8%)
Supraorbital ethmoid cell, n (%)	61 (34.8%)
Concha bullosa, n (%)	58 (33.1%)
Frontal recess cells, n (%)	37 (21.1%)

SD = Standard deviation.

**Table 2:** Interobserver Reliability (ICC Values).

Parameter	ICC	95% CI	*p*-Value
Ethmoid roof width	0.91	0.88–0.94	<0.001
Lateral lamella depth	0.87	0.83–0.90	<0.001
AP roof length	0.89	0.85–0.92	<0.001
Lateral lamella angle	0.94	0.92–0.96	<0.001
AEA–LL distance	0.90	0.87–0.93	<0.001

ICC = Intraclass correlation coefficient; CI = Confidence interval; AP = Anterior–posterior; AEA = Anterior ethmoidal artery; LL = Lateral lamella.

**Table 3:** Ethmoid Roof and Lateral Lamella Measurements (350 sides).

Parameter	Right Mean ± SD	Left Mean ± SD	*p*-Value
Ethmoid roof width (mm)	17.4 ± 2.8	17.1 ± 2.9	0.214
Lateral lamella depth (mm)	5.8 ± 1.6	5.7 ± 1.7	0.482
AP roof length (mm)	12.6 ± 2.3	12.4 ± 2.2	0.331
Lateral lamella angle (°)	33.8 ± 6.2	33.1 ± 6.5	0.289
AEA–LL distance (mm)	3.9 ± 1.4	4.0 ± 1.5	0.567

AP = Anterior–posterior; SD = Standard deviation; LL = Lateral lamella; AEA = Anterior ethmoidal artery.

**Table 4:** Keros Classification Distribution (350 sides).

Keros Type	n (%)
Type 1	86 (24.6%)
Type 2	203 (58.0%)
Type 3	61 (17.4%)

**Table 5:** Akcan Classification Distribution (350 sides).

Akcan Type	n (%)
Type 1—In-canal	140 (40.0%)
Type 2—Partially suspended	146 (41.7%)
Type 3—Fully suspended	64 (18.3%)

**Table 6:** Morphometric Comparison Across Akcan Types.

Parameter	Type 1 Mean ± SD	Type 2 Mean ± SD	Type 3 Mean ± SD	*p*-Value
Roof width (mm)	16.3 ± 2.4	17.9 ± 2.6	19.4 ± 2.7	<0.001
LL depth (mm)	5.2 ± 1.3	5.9 ± 1.4	6.8 ± 1.5	<0.001
LL angle (°)	31.4 ± 5.2	34.1 ± 5.6	37.6 ± 6.1	0.001
AP roof length (mm)	12.1 ± 2.0	12.7 ± 2.1	13.5 ± 2.3	0.004
AEA–LL distance (mm)	5.0 ± 1.2	3.8 ± 1.1	2.1 ± 0.9	<0.001
Supraorbital cell (%)	13.1%	36.3%	60.9%	<0.001

LL = Lateral lamella; AP = Anterior–posterior; SD = Standard deviation; AEA = Anterior ethmoidal artery. Bonferroni post hoc analysis demonstrated statistically significant differences between all pairwise Akcan type comparisons (Type 1 vs. Type 2, Type 1 vs. Type 3, and Type 2 vs. Type 3) for each continuous morphometric variable (all adjusted *p* < 0.05).

**Table 7:** Anatomical Variations Across Akcan Types.

Anatomical Variation	Type 1 (%)	Type 2 (%)	Type 3 (%)	*p*-Value
Septal deviation	38.5	36.9	40.6	0.531
Agger nasi cell	47.1	52.0	54.7	0.214
Concha bullosa	29.2	34.6	35.9	0.388
Frontal recess cell	18.5	22.1	23.4	0.472
Supraorbital ethmoid cell	13.1	36.3	60.9	<0.001

**Table 8:** Relationship Between Akcan and Keros Classifications.

Akcan Type	Keros 1 n (%)	Keros 2 n (%)	Keros 3 n (%)	*p*-Value
Type 1	58 (41.4%)	72 (35.5%)	10 (16.4%)	<0.001
Type 2	56 (40.0%)	85 (41.9%)	12 (19.7%)	
Type 3	22 (18.6%)	46 (22.6%)	39 (63.9%)	

**Table 9:** Pearson Correlation Analysis of Morphometric Parameters.

Variable Pair	Correlation (r)	*p*-Value
Roof width ↔ LL depth	0.41	<0.001
Roof width ↔ LL angle	0.29	0.002
LL depth ↔ AEA–LL distance	−0.36	<0.001
LL angle ↔ AEA–LL distance	−0.53	<0.001
AP roof length ↔ LL angle	0.28	0.004

LL = Lateral lamella; AP = Anterior–posterior; AEA = Anterior ethmoidal artery.

**Table 10:** Gender-Based Comparison of Ethmoid Roof and Lateral Lamella Morphometric Parameters.

Parameter	Male (n = 102) Mean ± SD	Female (n = 73) Mean ± SD	*p*-Value
Ethmoid roof width (mm)	17.3 ± 2.9	17.1 ± 2.7	0.412
Lateral lamella depth (mm)	5.8 ± 1.6	5.7 ± 1.5	0.538
AP roof length (mm)	12.6 ± 2.2	12.4 ± 2.1	0.461
Lateral lamella angle (°)	33.6 ± 6.4	33.2 ± 6.1	0.589
AEA–LL distance (mm)	3.9 ± 1.5	4.0 ± 1.4	0.621

**Table 11:** Multivariate Analysis Evaluating the Association Between Akcan Classification and Morphometric Parameters Adjusted for Keros Classification.

Variable	Odds Ratio (OR)	95% Confidence Interval	*p*-Value
Keros Type (per grade increase)	2.14	1.48–3.10	<0.001
Ethmoid roof width (per mm)	1.28	1.12–1.47	0.001
Lateral lamella depth (per mm)	1.41	1.18–1.69	<0.001
Lateral lamella angle (per °)	1.07	1.03–1.11	0.002
AEA–LL distance (per mm)	0.62	0.49–0.79	<0.001

## Data Availability

The data supporting the findings of this study are available from the corresponding author upon reasonable request. No publicly archived datasets were generated due to institutional privacy restrictions.

## Abbreviations

AEA	Anterior ethmoidal artery
AEA–LL distance	Distance between the anterior ethmoidal artery and the lateral lamella
AP	Anterior–posterior
CT	Computed tomography
ICC	Intraclass correlation coefficient
LL	Lateral lamella
LL angle	Lateral lamella inclination angle
LL depth	Lateral lamella depth
MPR	Multiplanar reconstruction
PACS	Picture Archiving and Communication System
SD	Standard deviation

## References

[1] Papadopoulou A.M., Chrysikos D., Samolis A., Tsakotos G., Troupis T. (2021). Anatomical Variations of the Nasal Cavities and Paranasal Sinuses: A Systematic Review. Cureus.

[2] Kaplanoglu H., Kaplanoglu V., Dilli A., Toprak U., Hekimoğlu B. (2013). An analysis of the anatomic variations of the paranasal sinuses and ethmoid roof using computed tomography. Eurasian J. Med..

[3] Vinciguerra A., Dohin I., Daloiso A. (2024). Iatrogenic Cerebrospinal Fluid Leak in Endoscopic Sinus Surgery: Topographical Map and Influence of Skull Base Asymmetry. J. Pers. Med..

[4] Alhumaid H., Alsowinea A., Alamer A. (2025). CT Analysis of Variations in the Medial Maxillary Wall Relative to the Medial Orbital Wall: Implications for Surgical Risk Stratification from an Endoscopic Perspective. Life.

[5] Mahdian M., Karbasi Kheir M. (2022). CBCT Assessment of Ethmoid Roof Variations through Keros, Gera, and TMS Classifications. Int. J. Otolaryngol..

[6] Keros P. (1962). On the practical value of differences in the level of the lamina cribrosa of the ethmoid. Z. fur Laryngol. Rhinol. Otol. und Ihre Grenzgeb..

[7] Kolak M., Kızılgöz V. (2024). Examination of ethmoidal roof regarding Keros and Yenigun classifications in a Turkish population: A computerized tomography study. Surg. Radiol. Anat..

[8] Ben-Shlomo N., Jayender J., Guenette J.P., Corrales C.E. (2023). Iatrogenic inner ear dehiscence associated with lateral skull base surgery: A systematic analysis of drilling injuries and their causal factors. Acta Neurochir..

[9] Hudelist B., Elia A., Roux A., Schumacher X., Hamza M., Paun L., Moiraghi A., Oppenheim C., Naggara O., Muto J. (2025). Management and outcomes of internal carotid artery, anterior cerebral artery, or middle cerebral artery injury during microsurgical approach of the anterior and middle cranial skull base: Insights from a systematic review and a case series. Neurosurg. Rev..

[10] Kho J.P.Y., Tang I.P., Tan K.S., Koa A.J., Prepageran N., Rajagopalan R. (2019). Radiological Study of the Ethmoidal Arteries in the Nasal Cavity and Its Pertinence to the Endoscopic Surgeon. Indian J. Otolaryngol. Head. Neck Surg..

[11] Yenigun A., Goktas S.S., Dogan R., Eren S.B., Ozturan O. (2016). A study of the anterior ethmoidal artery and a new classification of the ethmoid roof (Yenigun classification). Eur. Arch. Oto-Rhino-Laryngol..

[12] Gibelli D., Cellina M., Gibelli S., Floridi C., Termine G., Sforza C. (2022). Anatomical Variations of Anterior Ethmoidal Foramen and Cribriform Plate: Relations With Sex. J. Craniofacial Surg..

[13] Moon H.J., Kim H.U., Lee J.G., Chung I.H., Yoon J.H. (2001). Surgical anatomy of the anterior ethmoidal canal in ethmoid roof. Laryngoscope.

[14] Floreani S.R., Nair S.B., Switajewski M.C., Wormald P.J. (2006). Endoscopic anterior ethmoidal artery ligation: A cadaver study. Laryngoscope.

[15] Yang Y.X., Lu Q.K., Liao J.C., Dang R.S. (2009). Morphological characteristics of the anterior ethmoidal artery in ethmoid roof and endoscopic localization. Skull Base.

[16] Takeda T., Kajiwara R., Omura K., Otori N., Wada K. (2020). Analysis of anatomical variation of the inclination of lamellas attached to the skull base and its correlation with the anterior ethmoidal artery floating in the ethmoid sinus for use in endoscopic sinus surgery. Surg. Radiol. Anat..

[17] Abdullah B., Lim E.H., Mohamad H., Husain S., Aziz M.E., Snidvongs K., Wang Y., Musa K.I. (2019). Anatomical variations of anterior ethmoidal artery at the ethmoidal roof and anterior skull base in Asians. Surg. Radiol. Anat..

[18] Acar M., Şeker B., Uğur S. (2024). The Morphometric Analysis of the Ethmoid Roof for Endoscopic Sinus Surgery With Multidetector Computed Tomography. J. Craniofacial Surg..

[19] Asal N., Bayar Muluk N., Inal M., Şahan M.H., Doğan A., Arikan O.K. (2019). Olfactory Fossa and New Angle Measurements: Lateral Lamella-Cribriform Plate Angle. J. Craniofacial Surg..

[20] Aksoy D., Karagöz Y., Mahmutoğlu A.S. (2022). Ethmoid roof morphometric measurements of a pediatric population using computed tomography. Surg. Radiol. Anat..

[21] Wang H., Li A., Bie T., Dou X., Li Y., Xu Z., Li X., Chen Y., Li M., Wei X. (2025). Correlation Analysis of the Pneumatization of the Supraorbital Ethmoid Cell and the Position of the Anterior Ethmoidal Artery. Ear Nose Throat J..

[22] Jiang Y., Tang L., Ding J., Zhang Y., Zhang J. (2025). The CT image characteristics of anterior ethmoidal artery and its significance in nasal endoscopic surgery. J. Clin. Otorhinolaryngol. Head Neck Surg..

[23] Jang D.W., Lachanas V.A., White L.C., Kountakis S.E. (2014). Supraorbital ethmoid cell: A consistent landmark for endoscopic identification of the anterior ethmoidal artery. Otolaryngol.-Head Neck Surg..

[24] Cruz A.A.V., Cunha B.S. (2024). Position of the anterior ethmoidal foramen and trauma to the cranial base during transconjunctival medial orbital decompression: A systematic literature review. Orbit.

[25] Yousuf M., Jamil A., Hashmi Q., Hameed K., Sami M., Shaikh T., Zia S. (2024). Anatomical Variation of the Olfactory Fossa According to Keros and Yenigun Classifications in Karachi, Pakistan. Cureus.

[26] Randhawa L.S., Semwal A., Srivastava R.K., Hernot S. (2024). A Detailed Assessment of Variations of Ethmoid Roof, Olfactory Fossa, and Anterior Ethmoidal Artery on CT Scan of Paranasal Sinuses of 200 Patients. Indian. J. Otolaryngol. Head. Neck Surg..

[27] Muñoz-Leija M.A., Yamamoto-Ramos M., Barrera-Flores F.J., Treviño-González J.L., Quiroga-Garza A., Méndez-Sáenz M.A., Campos-Coy M.A., Elizondo-Rojas G., Guzmán-López S., Elizondo-Omaña R.E. (2018). Anatomical variations of the ethmoidal roof: Differences between men and women. Eur. Arch. Oto-Rhino-Laryngol..

[28] Dassi C.S., Demarco F.R. (2020). The Frontal Sinus and Frontal Recess: Anatomical, Radiological and Surgical Concepts. Int. Arch. Otorhinolaryngol..

[29] Al Habsi T., Al-Ajmi E., Washahi M.A., Lawati M.A., Maawali S.A., Mahajan A., Sirasanagandla S.R. (2024). Does Frontal Recess Cell Variation Associate with the Development of Frontal Sinusitis? A Narrative Review. Diagnostics.

[30] Sasmal D.K., Singh M., Nayak S., Panda S., Reddy P.N. (2022). Lateral Lamella of Cribriform Plate (LLCP): A Computed Tomography Radiological Analysis. Indian. J. Otolaryngol. Head. Neck Surg..

[31] Özdemir A., Bayar Muluk N. (2022). The important adjacent structures for anterior ethmoidal artery in FESS: Anterior ethmoidal artery canal angle, supraorbital ethmoid cells and Keros classification. J. Clin. Neurosci..

[32] Preti A., Mozzanica F., Gera R., Gallo S., Zocchi J., Bandi F., Guidugli G., Ambrogi F., Yakirevitch A., Schindler A. (2018). Horizontal lateral lamella as a risk factor for iatrogenic cerebrospinal fluid leak. Clinical retrospective evaluation of 24 cases. Rhinology.

[33] Baban M.I.A., Hadi M., Gallo S., Zocchi J., Turri-Zanoni M., Castelnuovo P. (2017). Radiological and clinical interpretation of the patients with CSF leaks developed during or after endoscopic sinus surgery. Eur. Arch. Oto-Rhino-Laryngol..

[34] Özdemir A., Bayar Muluk N., Bekin Sarikaya P.Z., Yilmazsoy Y. (2023). Supraorbital ethmoid cells (SOECs), anterior ethmoid artery notch and ethmoid roof relation in PNSCT. J. Clin. Neurosci..

[35] Yüksel Aslier N.G., Karabay N., Zeybek G., Keskinoğlu P., Kiray A., Sütay S., Ecevit M.C. (2017). Computed Tomographic Analysis: The Effects of Frontal Recess Morphology and the Presence of Anatomical Variations on Frontal Sinus Pneumatization. J. Craniofacial Surg..

